# P-418. Do We Need Broad Empirical Antibiotics for Upper Urinary Tract Infections Caused by Chromosomal AmpC-Producing Enterobacterales in Children?

**DOI:** 10.1093/ofid/ofaf695.635

**Published:** 2026-01-11

**Authors:** Nobuhiro Kanie, Yuto Otsubo, Meiwa Shibata, Yuho Horikoshi

**Affiliations:** Tokyo Metropolitan Children’s Medical Center, Musashino shi, Tokyo, Japan; Tokyo Metropolitan Children's Medical Center, Fuchu, Tokyo, Japan; Tokyo Metropolitan Children's Medical Center, Fuchu, Tokyo, Japan; Tokyo Metropolitan Children's Medical Center, Fuchu, Tokyo, Japan

## Abstract

**Background:**

The emergence of antimicrobial resistance has complicated management of infections. We investigated whether empirical therapy discordant for chromosomal AmpC-Producing Enterobacterales (AmpC-E) had a clinical impact before switching to definitive therapy in pediatric urinary tract infections (UTIs).

**Figure:**
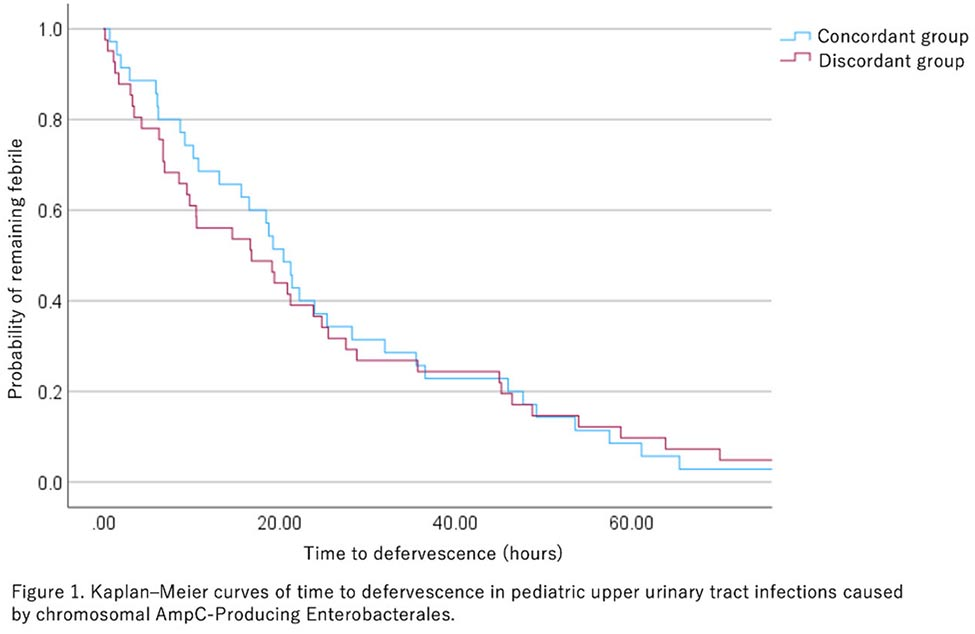

**Methods:**

A retrospective study was conducted at Tokyo Metropolitan Children’s Medical Center from July 2010 to January 2024. Inclusion criteria were patients aged < 16 years with fever ≥ 38.0℃ and ≥ 10⁴ CFU/mL of AmpC-E in urine cultures. Exclusion criteria were pre-antibiotic defervescence, alternative diagnoses, or polymicrobial growth ( ≥ 3 species). Patients were classified into two groups. The discordant group received initial antibiotics considered ineffective for AmpC-E, such as penicillins or first to third generation cephalosporins. The concordant group received antibiotics generally recommended for AmpC-E, such as aminoglycosides or fourth generation cephalosporins or carbapenems. The primary outcome was time to defervescence. The secondary outcome was 30-day culture confirmed recurrence. Time to defervescence was analyzed by Kaplan–Meier curves.

**Results:**

Of 135 cases identified, 76 met the inclusion criteria. The discordant and the concordant groups were 41 and 35, respectively. Median age was 6 months (IQR 4.8–16.8) in the discordant group and 8.4 months (IQR 4.8-33.6) in the concordant group. Common pathogens included *Enterobacter cloacae* (30%) and *Klebsiella aerogenes* (26%). Catheter-associated UTIs were 33%. Median time to defervescence was 16.9 hours (95% CI 5.8–28.0) in the discordant group and 20.5 hours (95% CI 17.1–23.9) in the concordant group, with no significant difference (*p* = 0.998) (Figure 1). 30-day culture confirmed recurrence occurred in one case each group.

**Conclusion:**

In pediatric UTIs caused by AmpC-E, initial discordant therapy was not associated with adverse clinical outcomes, suggesting that unnecessary use of broad-spectrum antibiotics may be avoided in empirical treatment.

**Disclosures:**

All Authors: No reported disclosures

